# Independent influence of age on heart rate recovery after flywheel exercise in trained men and women

**DOI:** 10.1038/s41598-021-91565-w

**Published:** 2021-06-08

**Authors:** Damir Zubac, Nandu Goswami, Vladimir Ivančev, Zoran Valić, Boštjan Šimunič

**Affiliations:** 1 Science and Research Centre Koper, Institute for Kinesiology Research, Koper, Slovenia; 2 Faculty of Kinesiology, University of Split, Split Croatia, Croatia; 3 Gravitational Physiology, Aging and Medicine Research Unit, Physiology Division, Otto Loewi Center of Vascular Biology, Immunity and Inflammation, Medical University of Graz, Graz, Austria; 4 Department of Integrative Physiology, School of Medicine, University of Split, Split, Croatia

**Keywords:** Physiology, Health care

## Abstract

The present study examined whether differences in the heart rate recovery following flywheel exercise cessation were associated with differences in maximal oxygen uptake (${\dot{\text{V}}}$O_2_ max.), age and sex in trained adults. Eleven men (age range 22–49 years, ${\dot{\text{V}}}$O_2_ max. = 43.6 ± 7.6 mL kg min^−1^) and ten women (age range 20—53 years, ${\dot{\text{V}}}$O_2_ max. = 38.0 ± 5.7 mL kg min^−1^) were randomly assigned to complete a squat-exercise on the flywheel ergometer set at three different moments of inertia, while their cardiovascular responses were continuously monitored. During the flywheel exercise the mean arterial pressure rose by ~ 35 to 40% (*p* = .001), and the increment was more robust in men than women. The cardiac index was two-fold greater across both sexes compared to the baseline (*p* = .001), while the rise in heart rate (~ 144 bpm) was more pronounced in women to compensate for their load-dependent stroke index decline (*p* = .001). The load-independent time-course changes in heart rate recovery markers were comparable between the sexes. When these indicators were pooled, a stepwise regression revealed age as the only relevant predictor of both fast and slow components of the heart rate recovery (~ 30% of the shared variance explained, *p* = .014). The present data suggest that the heart rate recovery declines with age, irrespective of sex, or well-preserved cardiorespiratory fitness in moderately-trained adults.

## Introduction

In sports medicine and physiology, the heart rate (HR) recovery parameters are often evaluated to obtain a valid, non-invasive insight into cardiac autonomic nervous system (ANS) function across various populations^1–4^. In athletes, the HR recovery assessment is considered to be a viable tool to monitor overall fitness level, endurance performance adaptation, and overtraining syndrome development^1^. In clinical population, a slower HR recovery after exercise cessation is a strong predictor of various cardiovascular disease (CVD) development and all-cause mortality, since a delayed HR recovery indicates cardiac ANS impairment^2–4^. However, under resting conditions, the impairment of the cardiac autonomic function is sometimes less apparent^5^, and therefore exercise stress tests, such as the Bruce test, are performed to maximally stress and provoke the cardiovascular response. Recent systematic and narrative reviews by Fecchio et al.^3^ and Peçanha et al.^4^, summarized the clinical validity and diagnostic accuracy of various HR recovery indicators, with data collected primarily after aerobic exercise using both active and passive recovery methods. Specifically, extensive literature published over the last 20 years have unfolded evidence on HR recovery following aerobic exercise^6^, performed on a cycle ergometer^7,8^ or a treadmill^9^.

Interestingly, less is known regarding the cardiovascular strain during multiple joint, whole-body resistance exercise, despite a growing popularity of the resistance exercise among different populations, including women^10^. Many studies suggest that the flywheel, a gravity-independent resistance exercise model, is one of the most effective resistance exercise models. In fact, the flywheel exercise model is widely used to counter muscle atrophy, or to mitigate the risk of musculoskeletal injury occurrence^11^, a major concern in sports medicine. Aside from the previously mentioned benefits, flywheel ergometer is not completely free from risks. For instance, the mechanical overload during the eccentric phase of flywheel exercises is accompanied by greater activation and strain of the musculo-tendineous structures compared to traditional resistance training exercises^11–13^. Further, it is still unknown whether this greater mechanical load on the peripheral musculature induces additional demands on the cardiovascular system due to the mechanical occlusion of large blood vessels occurring throughout the entire range of motion of flywheel training, which could lead to a potentially greater systemic burden.

Previous work showed that conventional resistance training typically induces a massive cardiovascular burden, with average blood pressure (BP) reaching ~ 230/130 mmHg during the maximally loaded squat exercise^14,15^. Currently, established criteria for a comprehensive evaluation of cardiovascular dynamics response during and following flywheel resistance exercise are lacking. Hefferman et al.^16^ described the time-course changes in the cardiac ANS via heart rate variability (HRV) assessment following traditional resistance training, and suggested that resistance exercise imposes a greater stress on the cardiac ANS compared to aerobic exercise among men. Consequently, this is further translated into a greater reduction in cardiac parasympathetic tone during recovery. However, the current evidence on HR recovery following acute resistance exercise is limited to young^2^, or middle-aged sedentary male participants^8^, and less is known regarding the cardiovascular response and recovery following acute resistance intervention in women, especially taking into consideration their age and hormonal status^17^. In studies recruiting females, the HR recovery evaluation following exercise stress tests was not controlled for a specific cycle phase^18^, nor their oxygen uptake (${\dot{\text{V}}}$O_2_ max.) was determined^18,19^, and data were not separately analyzed from that of men^19^. Thus, data on HR recovery following various resistance exercise protocols in women have not yet been thoughtfully examined, which appears considerably important in light of the evidence reporting age and sex-based differences in BP response^17^, cardiac ANS response, and muscle sympathetic nerve activity at rest^20,21^ or during isolated submaximal contractions.

The aim of the present study was to examine whether differences in the HR recovery following the flywheel-exercise were associated with differences in the cardiorespiratory fitness profile (presented as ${\dot{\text{V}}}$O_2_ max.), sex and age among healthy trained adults. We hypothesized that the HR recovery would be sex-dependent, rather than load-dependent, while a higher aerobic fitness would modulate the HR recovery profile following flywheel exercise cessation.

## Methods

### Study population

Through social media advertisement and personal communication participants of both sexes were invited to volunteer in the present study. They reported to the laboratory for two preliminary screening visits, one week before the onset of experimental sessions. Exclusion criteria were: obesity (BMI ≥ 30 kg/m^−2^), smoking, arterial hypertension (≥ 140/90 mmHg), previous history of cardiovascular events, arrhythmias, previous history of neuromuscular injuries, metabolic diseases, supplement intake, drug medication, and contraceptive pill use. All participants (n = 21) gave their written informed consent to participate after being fully informed about the procedures and risks involved in agreement with the Declaration of Helsinki regarding Biomedical research involving human participants and was approved by the Republic of Slovenia National Medical Ethics Committee (120-487/2018/21).

### Study design

Participants (n = 21, 48% women) completed five laboratory visits, including two preliminary and three experimental sessions. During preliminary visits the participants were advised to refrain from vigorous exercise, caffeine, large meals or alcohol consumption during the 24 h prior to each experimental session. All female participants were tested in the early or mid-follicular phase of their menstrual cycle to minimize the potential effects of hormonal fluctuations on cardiovascular parameters, blood vessel compliance, and ANS function^22^. After the completion of the preliminary sessions, all participants were randomly assigned into three different experimental conditions; that is, the flywheel-squat exercise set at three different moments of inertia (or loads, with details explained below). During the first screening visit their medical histories, resting HR, BP, SpO_2_, %, and anthropometrics were measured. The PAR-Q questionnaire was used to obtain data on their physical activity level. On their second visit, participants were familiarized with the testing procedures, and their ${\dot{\text{V}}}$O_2_ max. was determined. In addition, all participants took part in one familiarization session on the flywheel ergometer in order to minimize learning effects. Throughout the experimental sessions all participants arrived in the laboratory at the same time of the day, rested quietly for 10 min while being instrumented with the Task force monitor finger-cuff, cardio-impedance electrodes, and electrocardiogram (ECG) simultaneously. The hand was inserted into a neoprene shoulder mitella to provide additional support and secure the positioning at the level of the heart during flywheel exercise. Blood pressure was constantly measured via photoplethysmograph, using a pneumatic cuff, instructed to be held at the level of the heart throughout the session^23^, cardio-impedance electrodes were used to estimate the stroke volume and the cardiac output, while the ECG readings provided insight into the HR for each participant. All signals were simultaneously recorded via Task Force Monitor (CNSystems, Graz, Austria). The experimental procedure typically lasted ~ 60 min. Following instrumentation, the recording of the experimental sessions started. Each experimental session was divided into five different phases, which required the participants to (1) rest quietly in a supine position for 10 min (including signal acquisition and actual data collection); (2) stand for 5 min; (3) complete a 5-min, standardized, warm-up stepping protocol with a metronome set at 95 beats per minute; (4) perform two consecutive vigorous squat exercises on the flywheel ergometer (interspersed with 2 min of rest in-between flywheel sessions, according to Lundberg et al.^13^; and, (5) immediately return to supine position for 5 min following the flywheel exercise cessation in agreement with the passive recovery protocol proposed by Morise^24^. Study participants completed all three experimental loads within one week, while each experimental procedure was separated by at least 48 h. To avoid the effects of confounding variables^25^, all physiological assessments were performed at a similar time of day (between 07:30 and 11:30 a.m.), in a closed and ventilated facility, with a temperature range of 20–22 °C, by the same researchers, using the same equipment throughout the investigation. All data were collected during February and March to control the effects of seasonal variations of BP.

### Preliminary assessments

The preliminary screening sessions were scheduled in the lab between ~ 07:30 and 08:30 a.m. The anthropometrics were determined via digital scale (Seca 769, Hamburg, Germany). After this, the participants were instructed to lie supine for 15 min, and a pneumatic cuff was positioned on their upper, non-dominant arm to assess resting BP via an automated sphygmomanometer (Dash 2000; General Electric, Milwaukee, WI, USA). In parallel, oxygen saturation and pulse frequency were simultaneously monitored using a pulse oximeter probe placed on the middle finger of the dominant arm.

### Measurements of ${\dot{\text{V}}}$O_2_ max

Peak power output, ${\dot{\text{V}}}$O_2_ max., and HR were determined in all participants via an incremental protocol on an electronically braked cycle-ergometer (Ergoline 900, Hamburg, Germany) that was synchronized with the cardiopulmonary exercise testing unit (Quark, Cosmed, Rome, Italy). Calibration of the metabolic analyzer was performed against two standard gas mixtures (room air, and 16.0% O_2_ and 5.0% CO_2_, respectively) prior to each cycle-ergometry session. A large 3-L syringe (model 5530, Hans Rudolph inc., KC, USA) was used to calibrate the turbine volume transducer, as specified by the manufacturer. All respiratory parameters were evaluated on a breath-by-breath basis, and HR was registered continuously with a Garmin monitor (HRM-3 SS, Kansas, USA). The protocol started at 20 W for 3 min, the participants instructed to maintain pedaling frequency of 70 rpm by continuously monitoring ergometer display during the protocol. Each three-second interval power output was increased by 1 W (20 W/min^−1^). The test was concluded when the participants reached voluntary exhaustion and/or pedaling frequency decreased by more than 5 rpm despite resilient cheering. After the protocol was completed, each subject cooled down for 5 min by performing unloaded pedaling. The ${\dot{\text{V}}}$O_2_ max. was established as the highest 20 s ${\dot{\text{V}}}$O_2_ determined via rolling average readings sampled during the last minute of the testing, while the peak power output and HR were defined as readings attained at the test cessation, as recently described in the literature^26^.

### Flywheel exercise

The flywheel-exercise bout(s) were performed on a commercially available ergometer (nHance, Barcelona, Spain) in a standing position, with both legs positioned on the ergometer set at low (0.025 kg m^2^), moderate (0.05 kg m^2^) and high (0.075 kg m^2^) levels of inertia. Following a standardized, stepping warm-up routine, all participants were positioned on the flywheel-ergometer with their knee joints fully extended in the starting position. Straps were placed around their chests and attached to the ergometer, in accordance with the manufacturer’s guidelines. After the initial wheel acceleration at exercise onset, the participants performed 2 sets of 7 repetitions of squat exercises on the ergometer with maximal effort (with 2 min of rest between both sets), as described in detail by Lundberg et al.^13^. The average concentric power was sampled at 1 kHz via the Chronopic friction encoder (Chronojump, Barcelona, Spain) for each repetition and later normalized per body mass of the participant. The friction encoder and its sensors were attached to the flywheel ergometer. Visual feedback of the power output was displayed to the researchers on a computer screen throughout the experimental procedure via Chronopic software.

### Continuous non-invasive cardiovascular assessment

To assess the BP response during experimental protocol, a finger photoplethysmograph and an upper arm sphygmomanometer were used. At the start of instrumentation, the participant sat down while a finger plethysmograph cuff was placed on the middle finger of the right arm at the level of the heart. The upper arm sphygmomanometer was positioned on the upper left arm. In parallel, the thoracic-impedance electrodes were positioned according to the manufacturer’s guidelines (CNSystems, Graz, Austria), to allow a non-invasive insight into the central hemodynamics throughout the experimental protocol. Briefly, the stroke volume and the cardiac output were continuously monitored, and normalized by the body surface area to obtain the participant-specific parameters, stroke index (SI) and cardiac index (CI). Next, the HR was obtained from the bipolar 3-lead ECG, placed according to previously established guidelines for Task force monitor instrumentation^27^. The power components of the RR interval (RRi) were defined as the time interval between the two R peaks in the ECG.

### Data processing and analysis

Raw data were visually inspected for ectopic and aberrant beats by two researchers independently and, if present, these beats were removed. Clean data were then exported from the TFM device as .txt files into an excel sheet and transferred into the Origin software (OriginLab Corp., Northampton, USA) for further analysis via customized functions. Specific time points (10-s time epochs) were used to characterize each different phase of the rest and exercise relevant for the statistical analysis: baseline (epoch 1 = 290–300 s); stepping warm-up (epoch 2 = 290–300 s); exercise 1 (epoch 3 = last 10 s); rest (epoch 4 = 60–70 s); exercise 2 (epoch 5 = last 10 s) as described in the literature^28,29^; while the post-exercise recovery data were analyzed as the mean value of each successive 30 s time-epoch during the entire 5 min of recovery, as recently proposed by Peçanha et al.^8^. Regarding the HR recovery indicators, these data were calculated from the absolute difference between the peak HR (mean HR, averaged over last 10 beats during exercise) and the HR values measured at 30, 60, and 300 s of recovery^8^. The mathematical modeling of the HR recovery time constant (τ) during the 5-min recovery was performed via a mono-exponential equation (the Levenberg–Marquardt least square regression), recently described by Bartels-Ferreira et al.^30^ to provide an individualized response for each participant.

1 $$ Y(t) = YBSLN + Amp\left( {1 - e ^{- \frac{t - TD}{\tau }}} \right) $$

### Statistics

Distribution normality was confirmed using the Shapiro–Wilk test, while the Q-Q plot was used to visually examine potential outliers. The GPower software (version 3.1.5) was used to determine the required sample size for this investigation. Student’s independent t-test was applied to establish differences between sexes in all dependent variables of interest. The effects of load (Low, Moderate, High) and time (Baseline, Warm-up, Exercise 1, Rest, Exercise 2) on different physiological parameters during the flywheel exercise (e.g., the cardiovascular response) were analyzed with a two-way ANOVA. Also, the HR recovery markers were entered into a two-way ANOVA, taking into accounting the effect of load (Low, Moderate and High) and sex as factors. For both models, in case a significant F-test was identified, a Bonferroni post-hoc was applied to determine multiple comparisons. During the recovery period time-course changes of all primary outcomes (cardiovascular parameters) were entered into a mixed linear model. In this model, the participants were depicted as random factors, while the moments of inertia (Low, Moderate and High load) and time-course changes were fixed factors. A forward-stepwise regression analysis was used to examine the association among HR recovery markers (HRR 60, 300 and τHRR) and different predictors including age, sex and ${\dot{\text{V}}}$O_2_ max. Coefficient of multiple determination, standard error of regression coefficient (SE(B)), together with standardized (b) and not standardized (β) regression coefficients were calculated. Multicollinearity of predictors was examined through the variance inflation factor. For all statistical analysis, type I error was set α = 5%, and further processed using data analysis software system Statistica ver. 13.5 (TIBCO software Inc., USA). All data are given as mean ± SD.

## Results

Twenty-one healthy, moderately-trained men and women completed all study procedures. Their baseline data are given in Table 1. The required study sample size was missed by one participant, according to the GPower analysis. On average, men were 14 cm taller and ~ 20 kg heavier than women (*p* = .001). There were no differences observed between sexes in resting cardiovascular parameters. For the cardiopulmonary exercise testing data, men had higher V_E_ and ${\dot{\text{V}}}$O_2_ max. (*p* = .01), while no differences were observed in their peak power output (W kg^−1^) and HR max. attained.

**Table 1:** Characteristics of the participants included.

	Men (n = 11)	Women (n = 10)	*p *value
**Anthropometrics**			
Age, y	34 ± 10	34 ± 11	.323
Body height, cm	185 ± 6	171 ± 4	.001
Body mass, kg	85.7 ± 10.3	65.9 ± 8.5	.001
BMI, kg m^−2^	25.1 ± 2.35	22.7 ± 2.6	.034
**Cardiovascular parameters**			
Resting HR, bpm	64 ± 5	69 ± 9	.132
Resting SBP, mmHg	118 ± 13	114 ± 9	.468
Resting DBP, mmHg	69 ± 9	65 ± 8	.307
Resting MAP, mmHg	86 ± 7	82 ± 8	.229
Resting SaO_2_, %	98.8 ± .9	98.8 ± .8	.106
**Cardiorespiratory profile**			
${\dot{\text{V}}}$_E_ (L min^−1^)	137 ± 23	102 ± 22	.002
${\dot{\text{V}}}$O_2_ max (mL kg min^−1^)	43.6 ± 7.6	38.0 ± 5.7	.001
RER,	1.21 ± .06	1.28 ± .08	.039
HR max. (bmp)	180 ± 8	183 ± 9	.200
HR reserve (bpm)	113 ± 3	114 ± 3	.571
PPO (W kg^−1^)	3.4 ± .5	3.2 ± .4	.266

*BMI* body mass index, *HR* heart rate, *SBP* systolic blood pressure, *DBP* diastolic blood pressure, *MAP* mean arterial pressure, *SaO*_*2*_* (%)* oxygen saturation, ${\dot{\text{V}}}$_*E*_ peak pulmonary ventilation, ${\dot{\text{V}}}$*O*_*2*_* max*. maximal oxygen uptake, *RER* respiratory exchange ratio, *HRmax*. maximal heart rate, *PPO* peak power output; Data are presented as mean ± SD.

Regarding the neuromuscular data during flywheel-exercise, all participants completed the flywheel-exercise within 13.6 ± 0.05 s, with no differences between the two consecutive exercise bouts duration (*p* = .284) or power output (*p* = .143). The power output was higher in men than women (12.3 ± 0.6 W kg^−1^ vs. 7.9 ± 0.05 W kg^−1^; *p* =  .01), and inversely proportional to the moments of inertia increment throughout.

**Figure 1:**
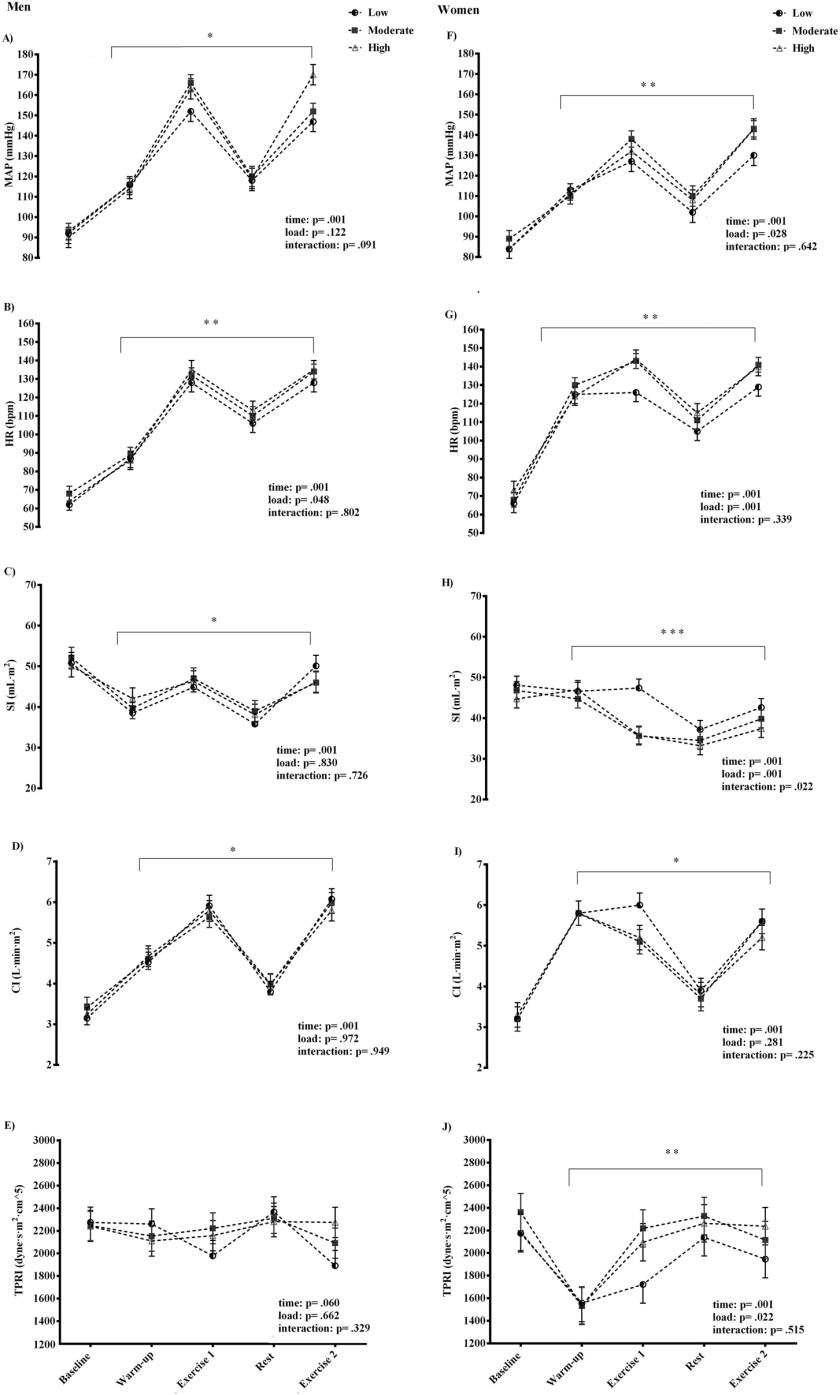
Cardiovascular response to the flywheel exercise. *MAP* mean arterial pressure, *HR* heart rate, *SI* stroke index, *CI* cardiac index, *TPRI* total peripheral resistance index. *Time effect. **Load effect. ***Interaction effect.

Data on cardiovascular response to three different flywheel-exercise loadings are given for both men and women in Fig. 1. The flywheel exercise caused an excessive rise in the mean arterial pressure (MAP) during all three experimental trials and across both exercise bouts, compared to baseline (time effect: *p* = .001). This increase was more robust in men than women. The CI was two-fold greater during exercise compared to baseline (*p* = .001) in both men and women, while a load-specific decline in the SI was observed in women alone (*p* = .001). Also, the total peripheral resistance response to exercise was load-specific in women (*p* = .022), but not in men. The HR readings during exercise were time and load dependent (*p* = . 01), with marginally higher HR readings in women (~ 144 bmp), compared to those reached by men (~ 140 bpm).

**Figure 2:**
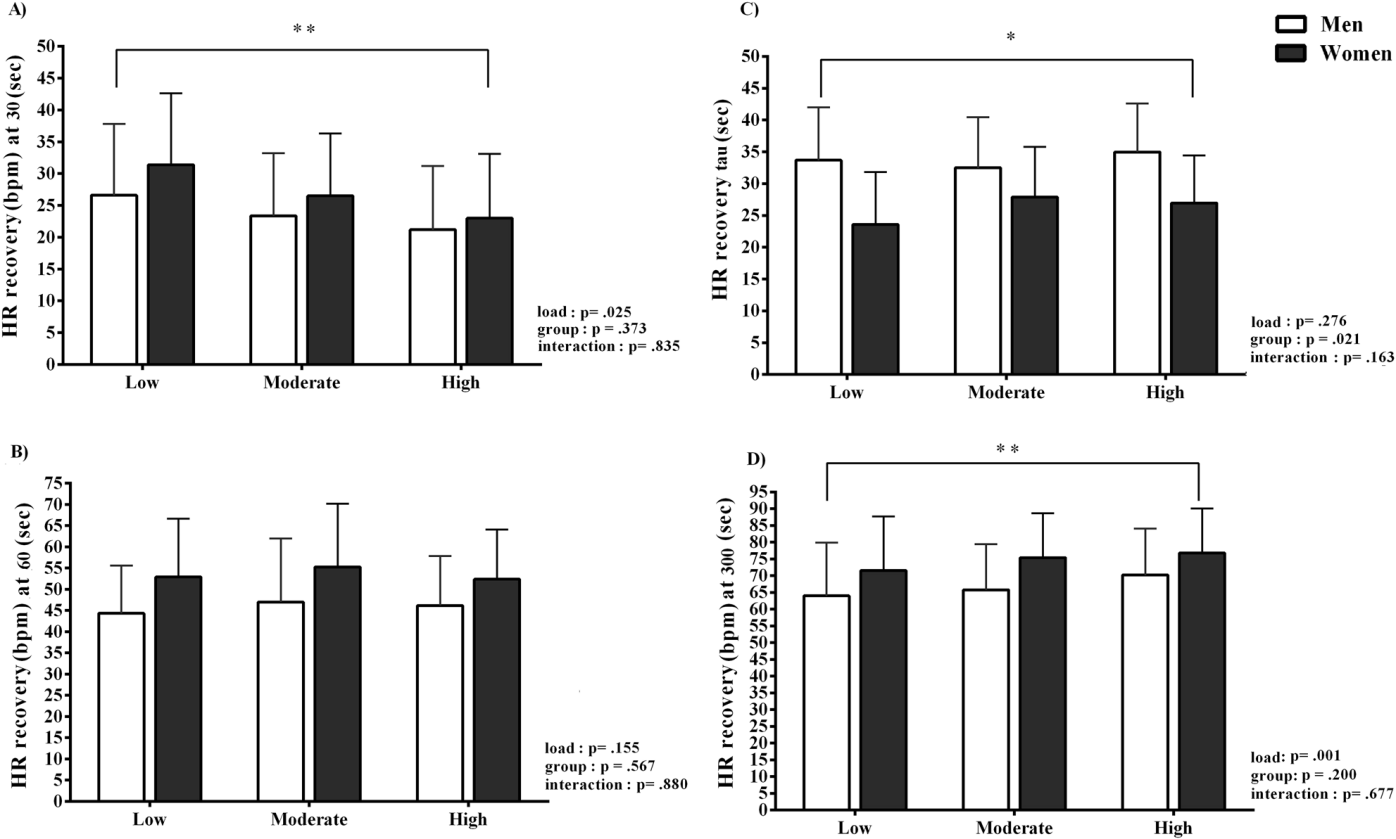
Heart rate recovery after the flywheel exercise. *HR recovery* heart rate recovery readings measured at 30, 60, and 300 s of recovery, *τHRR* time constant. *Group effect. **Load effect.

Data on four different HR recovery markers are displayed in Fig. 2. There were no interaction effects observed among the three different flywheel exercise loadings and sex, including HRR30 (*p* =  .835), HRR60 (*p* = .880), HRR300 (*p* = .677), and τHRR (*p* =  .163), suggesting load-independent time-course changes in HR recovery profile among men and women. Nevertheless, the main effects of load and sex were observed in HRR30 and HRR300 (load effect *p* =  .001) and τHRR (sex: *p* = .001) was shorter compared to women.

**Figure 3:**
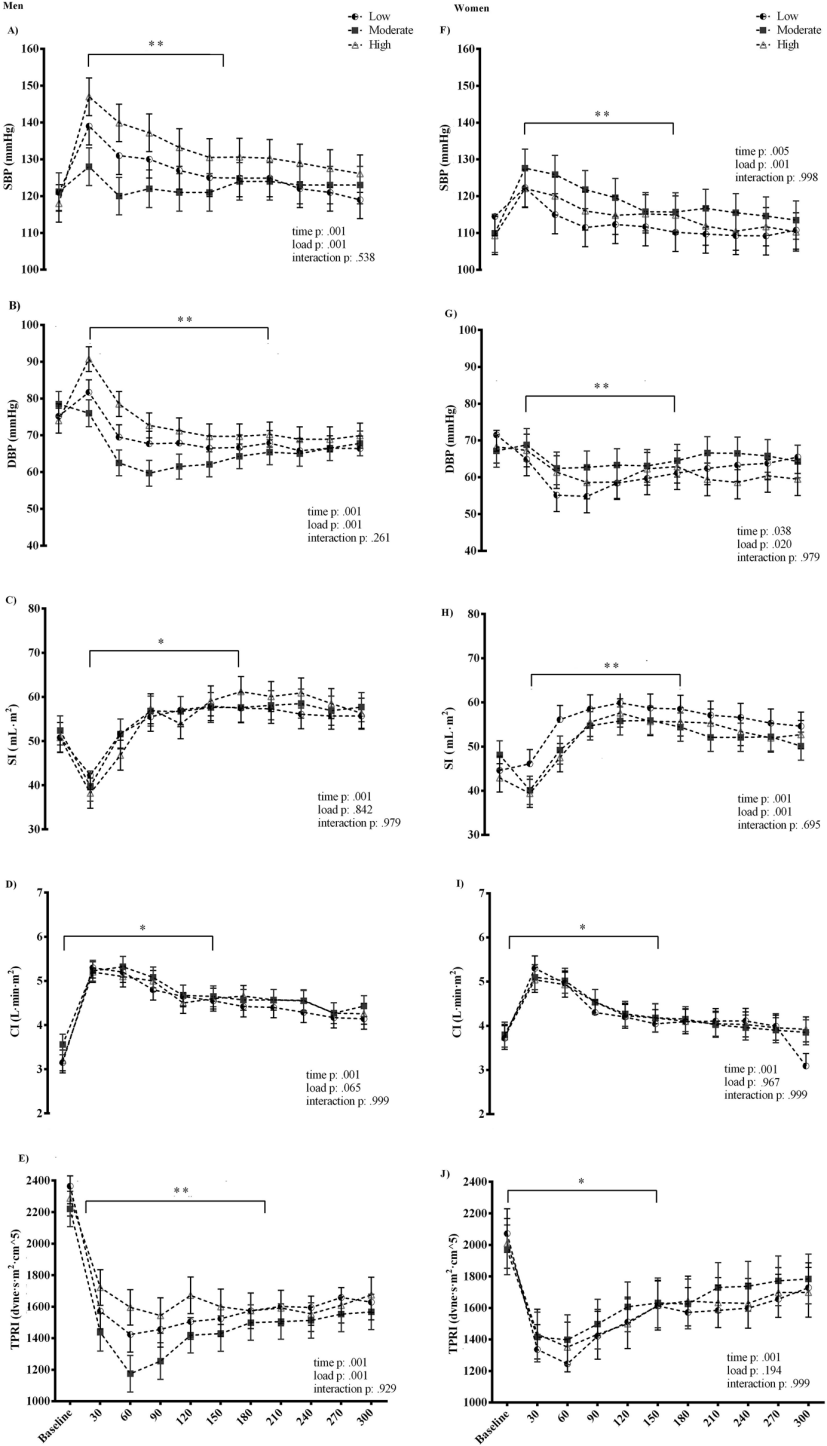
Cardiovascular response after the flywheel exercise with different load. *SBP* systolic blood pressure, *DBP* diastolic blood pressure, *SI* stroke index, *CI* cardiac index, *TPRI* total peripheral resistance index. *Time effect. **Load effect. ***Interaction effect.

Data on cardiovascular response during recovery are given in Fig. 3. The time-course changes in the cardiovascular response during recovery followed a similar, time-dependent pattern, which was more robust in men than women. In general, a gradual decrease was observed for the systolic, diastolic blood pressure, CI and total peripheral resistance, while the SI volume increased, indicating appropriate re-adjustments of the cardiovascular system during recovery.

**Figure 4:**
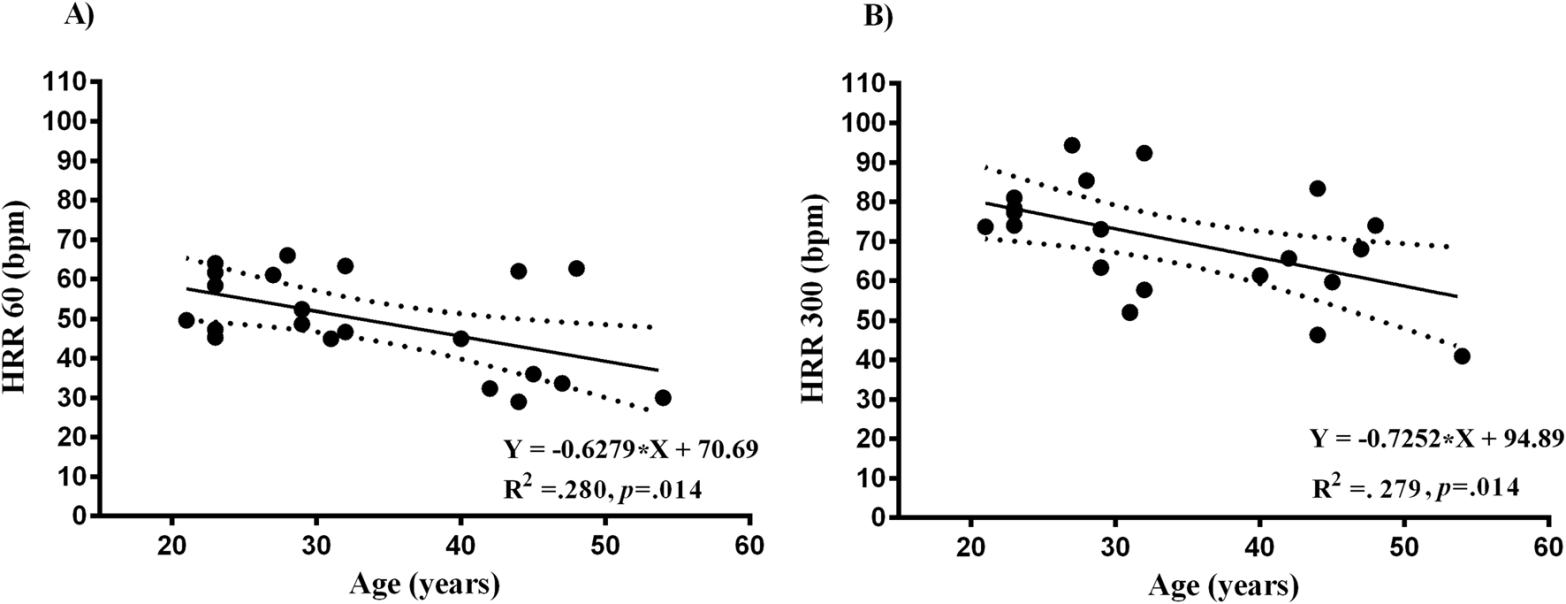
Multiple regression analysis of the HR recovery parameters. Heart rate recovery readings measured after (**A**) 60, and (**B**) 300 s versus the age of the participants**.**

Figure 4 reveals that in both regression models (HRR60, HRR300) the only independent predictor of the HR recovery was age. Indeed, the stepwise regression displayed that age alone explained ~ 30% (*p* = .014) of the shared variance of both HRR60 and HRR300, but not of τHRR (*p* = .915). It is important to underline that the forward stepwise model, the ${\dot{\text{V}}}$O_2_ max., was removed, as it was found to be an insignificant predictor (β = 0.239; *p* = .290 and β = 0.095; *p* = .680) of the HRR60 and HRR300, respectively. As expected, the multicollinearity of the predictors was not present in the model, since variance inflation factors ranged from 1.004 to 1.011 whilst the model tolerance ranged from 0.998 to 0.999. The supplementary material shows τHR recovery mono-exponential curve, with the overall goodness of fit (R^2^ = 0.93 ± 0.04, ranging from 0.81 to 0.98).

## Discussion

The present study is the first to investigate the cardiovascular response during flywheel resistance exercise and recovery in healthy, moderately-trained participants of both sexes. Overall, flywheel-training induced a substantial and excessive response of the cardiovascular system.

Briefly, the flywheel-exercise stimulus at different levels of inertia created a substantial, challenge to the cardiovascular system. An excessive rise in MAP and CI were observed throughout all flywheel-loads, with MAP readings reaching as high as ~ 165 and ~ 145 mmHg during the highest exercise load in men and women, respectively. The CI was two-fold greater in both groups, compared to baseline (Fig. 1). This forceful muscle loading during flywheel squat-exercise, completed within ~ 12 to 14 s, triggered a load-specific SI reduction, markedly in women, likely due to the large mechanical and intrathoracic pressure on the vasculature generated throughout exercise. To compensate and maintain appropriate central hemodynamics during the highest flywheel-exercise set at 0.075 kg m^2^, the HR rose up to ~ 144 bpm in women (on average ~ 5 beats higher than men, Fig. 1). These findings corroborate well with recent work of Trinity et al.^17^, who studied the influence of sex and age on BP response during isolated lower-limb contractions. They found an excessive in BP, especially among aging, sedentary women, primarily due to the age-related changes of the stroke volume during submaximal plantar flexion exercise. When compared to the whole-body squat exercise, the rate of cardiovascular strain reported here is in line with the work of Lovell et al.^14^ and Zubac et al.^15^. However, our work provides a novel insight into the non-invasive cardiovascular response via simultaneous measurement of photoplethysmography, cardio-impedance electrodes and ECG, and overcomes the methodological shortcomings associated with photoplethysmography-based assessments alone^14,15^. Further, the flywheel exercise, unlike other whole-body resistance exercise routines, evades certain technical limitations of the non-invasive cardiovascular measurement by allowing the finger-cuff to be positioned at a fixed level of the heart to provide an exceptional opportunity to study cardiovascular demands during resistance exercise. Thus, our data add novel insights to the sparse literature on the non-invasive assessment of the cardiovascular responses during high-intensity resistance exercise, especially in trained women. Indeed, previous work^14,15^ was limited to men, reported only HR and BP data, and failed to provide any deeper insight into the complex cardiovascular adjustments during vigorous resistance exercise.

After exercise cessation the HR recovery markers, of both slow and fast recovery phases, were not affected by the flywheel-load manipulations, nor the sex of the participants (Fig. 2). This suggests that the adjustments of HR recovery markers, during fast HR recovery (indicated by the HRR30 and HRR60, markers of parasympathetic reactivation), and slow phase (the HRR300 and τHRR markers), were similar among the flywheel-loads and sexes (Fig. 2). More precisely, a comparable, load-independent HR recovery time-course change was also previously described by Imai et al.^31^ and later confirmed in the work of Lamberts et al.^19^. The novelty of the present study was that, for the first time, data on HR recovery pattern after the flywheel-load manipulations were investigated in moderately-trained women, tested in a specific cycle phase, as previous studies mostly involved male participants. Still, it is rather challenging to extend the present findings to those previously published in the literature. Previous work suggested that exercise model—aerobic exercise^8,9^, recovery protocols—standing vs. supine recovery^8^ and sex^17,21^ all influence the cardiac ANS function and subsequently the HR recovery pattern. In fact, a large body of previous work assessed the HR recovery after predominantly aerobic exercise (via cycling or running), and generally reported data on young and middle-aged men alone^8,9^. Here, we reported a HR recovery after a vigorous, all-out exercise bout, with an average power output of ~ 11 W kg^−1^ in men and ~ 9 W kg^−1^ in women. Present findings on HR recovery profile (absolute values) are slightly higher than data reported by Maeder et al.^9^, who detected ~ 32 beats at HRR60 following maximal effort running and cycling in healthy male participants. A valid explanation of differences observed between our results and those reported by Maeder et al.^9^ relates to differences in exercise models and recovery protocol characteristics, since his work, in contrast to ours, was focused on aerobic exercise and standing HR recovery evaluation. Similar readings at HRR60 (~ 30 beats) were reported by Peçanha et al.^8^, who studied the effects of metaboreflex sensitivity on HR recovery following 30 min cycling exercise at moderate intensity (70% of the individual ${\dot{\text{V}}}$O_2_ max.) in sedentary men (${\dot{\text{V}}}$O_2_ max. ~ 25 mL kg min^−1^). Peçanha et al.^8^, monitored the HR recovery in a standing position and suggested that the HR recovery was delayed via metaboreflex activation (manipulated via circulatory occlusions of the lower limbs) after 30 min cycling exercise. Alternatively, we assessed the HR recovery during supine rest, following two consecutive bouts of vigorous flywheel-squat exercise (interspersed with 2-min rests). When combined, the differences in the exercise and recovery type may explain the differences between our work and that of Peçanha et al.^8^. Firstly, the HR recovery in our study was likely influenced via baroreflex loop activation due to the postural modifications^32^, secondly, the ${\dot{\text{V}}}$O_2_ max. data may also explain the differences between the studies. Participants here had a ~ 40% higher ${\dot{\text{V}}}$O_2_ max., compared to previous findings of Peçanha et al.^8^, and it is well-accepted that the superior ${\dot{\text{V}}}$O_2_ max. profile modulates the ANS function and subsequently influences the HR recovery profile. Lastly, data presented here add to the sparse literature on the dynamics of cardiovascular recovery in moderately-trained women, as their overall HR recovery profile after the flywheel-resistance exercise cessation resembles data reported in men, and the findings of regression analysis (Fig. 4) were not influenced by the sex of the participants included.

Mean ${\dot{\text{V}}}$O_2_ max. readings observed here were ~ 20 to 25% higher compared to referent values of middle-aged men and women presented by Kaminsky et al.^33^ in a large epidemiological study looking at the aerobic fitness in general population. Across the literature, it is widely accepted that aging and lower ${\dot{\text{V}}}$O_2_ max. negatively affects the cardiac ANS function, and subsequently influences the HR recovery profile. Voss et al.^34^ reported that aging is instrumental to a vagal tone decline, while Buchheit and Ginder^35^ suggested that a superior ${\dot{\text{V}}}$O_2_ max. is positively associated with the enhanced sympathovagal balance (as determined from the HF/LF index, with ~ 25% of shared variance), in young active men of varying ${\dot{\text{V}}}$O_2_ max. Based on the above-mentioned, we anticipated that a superior ${\dot{\text{V}}}$O_2_ max. would protect against the age-dependent HR recovery decline. Also, our previous findings suggested that age-related differences did not attenuate the incremental cycling capacity/performance or ${\dot{\text{V}}}$O_2_/HR kinetics in moderately-fit women^26^. However, in that study the HR max. reached during cycle-ergometry was 12 beats lower in middle-aged compared to young women. Here, contrary to our initial hypothesis, age was found to be the most significant, independent predictor of the HR recovery profile during both fast and slow HR recovery phases (Fig. 4). We observed an inverse association between age and both HRR60 and HRR300, with ~ 30% of the shared variance among the latter variables. The present findings imply that middle-aged individuals, as they get older, tend to have delayed HR recovery, during both recovery phases, irrespective of their current ${\dot{\text{V}}}$O_2_ max. or sex. These findings seem to in line with the 20-year follow-up study on an elite Olympic medal winner^36^. Briefly, Nybo and co-authors^36^ observed that the HR max. of the above-mentioned, 5-time Olympic medal winner, had gradually decreased by ~ 20 beats over the course of his 20-year-long competitive career (data collection started when he was 19 y old), irrespective of a well-preserved aerobic fitness (his ${\dot{\text{V}}}$O_2_ max. was ~ 70 mL kg min^−1^ throughout) and performance capacity in parallel. Therefore, these data fit well into the healthy aging paradigm, and suggest that a well-preserved aerobic fitness level is not necessarily linked to the HR recovery adjustments over time. Our findings are further supported by the meta-analysis of Tanaka et al.^37^, performed on > 18,000 healthy men and women, where the authors showed that the HR max. declines throughout lifespan by 0.7 bpm year^−1^, irrespective of sex or ${\dot{\text{V}}}$O_2_ max. Regarding the age-related ANS function and its relation to HR recovery, recent epidemiological findings of Voss et al.^34^ showed that the ANS function (assessed via HRV time and frequency domain analysis) in ~ 2000 participants of both sexes is modified with advancing age—due to the progressive decline in HRV parameters, even in middle-aged groups (age range from 45 to 55) compared to their younger counterparts (age range from 35 to 44). These notions are further reinforced by work of Ferrari et al.^38^ who reported detrimental changes in the ANS function even in middle-age populations, which were primarily associated with cardiovascular and hormonal disturbances, including the loss of sinoatrial pacemaker cells and an increase in the concentration of circulating vasopressin^39^.

## Conclusion

In conclusion, the present study showed a similar, load-independent HR recovery profile in moderately-active middle-aged men and women, respectively. The HR recovery markers were found to decline with age, irrespective of the ${\dot{\text{V}}}$O_2_ max. or sex of the participants included here. Interestingly, men had a more robust cardiovascular response to the flywheel during both exercise and recovery, compared to women. Thus, exercise interventions should be carefully tailored according to sex-based differences in BP response to flywheel exercise observed here, to minimize the likelihood of cardiovascular complications during vigorous attempts to overcome muscle-atrophy. Importantly, individuals practicing greater power outputs during flywheel exercise should keep in mind that acute high-intensity resistance exercise creates a massive cardiovascular burden, and typically results in reduction in cardiac parasympathetic tone during recovery. To ensure that exercise interventions can be safely prescribed for different populations, future studies should examine in more detail the interplay between muscle loads at different intensities and the subsequent sex and age-specific cardiovascular response during exercise and recovery (Supplementary Information 1).

## Supplementary Information

Supplementary Information 1.
