# Supplementary material for: Independent influence of age on heart rate recovery after flywheel exercise in trained men and women
Source: Sci Rep. 2021 Jun 8;11:12011. doi: 10.1038/s41598-021-91565-w (PMC8187710; doi:10.1038/s41598-021-91565-w)

**Independent Influence of Age on Heart Rate Recovery after Flywheel Exercise in Trained Men and Women**

Damir Zubac^1,2^, Nandu Goswami^3^, Vladimir Ivančev^2^, Zoran Valić^4^, and Boštjan Šimunič^1^

^1^Science and Research Centre Koper, Institute for Kinesiology Research, Koper, Slovenia

^2^University of Split, Faculty of Kinesiology, Split Croatia, Croatia

^3^Gravitational Physiology, Aging and Medicine Research Unit, Physiology Division, Otto Loewi Center of Vascular Biology, Immunity and Inflammation, Medical University of Graz, Graz, Austria

^4^University of Split, School of Medicine, Department of Integrative Physiology, Split, Croatia

**Leading author**: Prof. Nandu Goswami, MD, PhD

Supplementary material:

The supplementary material shows τHR recovery mono-exponential curve of one male (A) and one female B) participant, with the overall goodness of fit (R^2^ = .93±.04, ranging from .81 to .98).


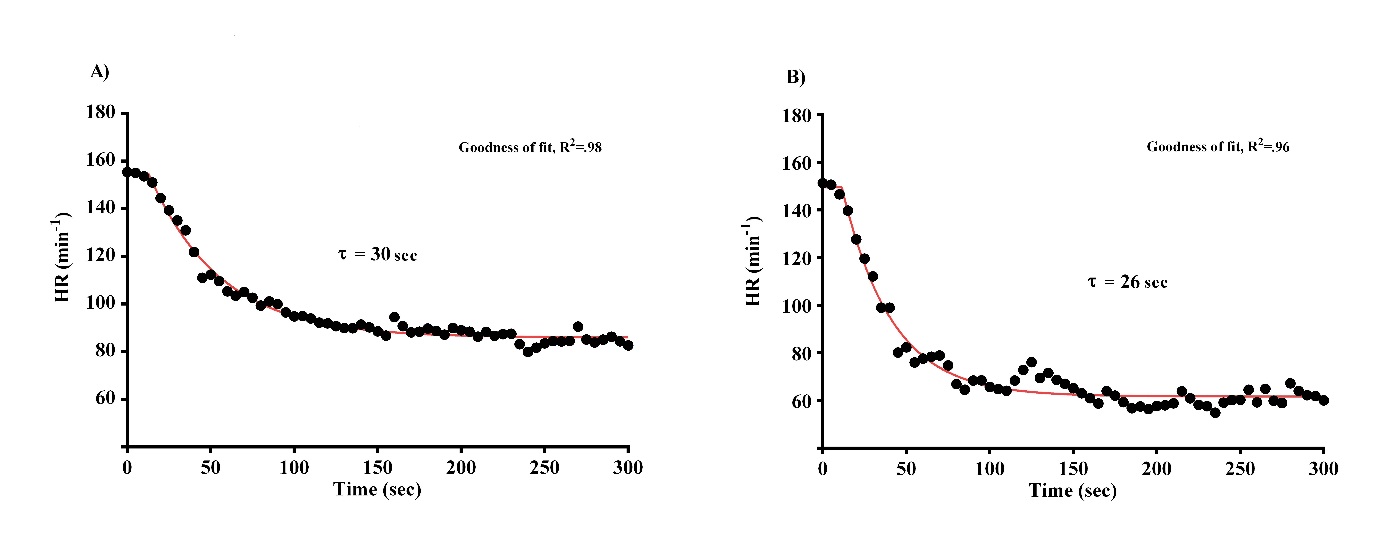

Supplement: Supplementary file 1 — Supplementary Information 1. [file 41598_2021_91565_MOESM1_ESM.docx]
